# Tackling an emerging epidemic: the burden of non-communicable diseases among people living with HIV/AIDS in sub-Saharan Africa

**DOI:** 10.11604/pamj.2020.36.271.22810

**Published:** 2020-08-12

**Authors:** Dunstan Achwoka, Regina Mutave, Julius Otieno Oyugi, Thomas Achia

**Affiliations:** 1 University of Nairobi, Institute of Tropical and Infectious Diseases (UNITID), College of Health Sciences, Nairobi, Kenya,; 2 Department of Medical Microbiology and Infectious Diseases, University of Manitoba, Winnipeg, Canada,; 3 School of Public Health, University of the Witwatersrand, Johannesburg, South Africa

**Keywords:** HIV, non-communicable diseases, sub-Saharan Africa

## Abstract

Sub-Saharan Africa (SSA) is at a crossroad. Over the last decade, successes in the scale up of HIV care and treatment programs has led to a burgeoning number of people living with HIV (PLHIV) in care. At the same time, an epidemiologic shift has been witnessed with a concomitant rise in non-communicable diseases (NCD) related morbidity and mortality. Against low levels of domestic financing and strained healthcare delivery platforms, the NCD-HIV syndemic threatens to reverse gains made in care of people living with HIV (PLHIV). NCDs are the global health disruptor of the future. In this review, we draw three proposals for low and middle-income countries (LMICs) based on existing literature, that if contextually adopted would mitigate against impending poor NCD-HIV care outcomes. First, we call for an adoption of universal health coverage by countries in SSA. Secondly, we recommend leveraging on comparably formidable HIV healthcare delivery platforms through integration. Lastly, we advocate for institutional-response building through a multi-stakeholder governance and coordination mechanism. Based on our synthesis of existing literature, adoption of these three strategies would be pivotal to sustain gains made so far for NCD-HIV care in SSA.

## Essay

Non-communicable diseases (NCDs) are the leading cause of death globally. In 2016 alone, of the world’s 57 million deaths, NCDs contributed to 41 million (71%) [1]. Four NCDs in particular - cardiovascular diseases (including hypertension, heart attack and stroke), cancer, chronic respiratory diseases and diabetes mellitus made the largest contribution to both morbidity and mortality. Low and middle-income countries (LMICs), that also carry a significant burden of HIV/AIDS, bore a disproportionately higher mortality burden. LMICs contributed to 78% of all NCD deaths and 85% of premature deaths [2]. Home to about 19 million (52%) of the 37 million PLHIV [3], sub-Saharan Africa (SSA) has a predominantly generalized HIV/AIDS epidemic. Overall, general population have a low HIV prevalence compared to key population who contribute up to 47% of new infections. Key populations are defined as groups who due to specific higher-risk behaviors are at an increased risk of HIV. Among others, key population groups include: men who have sex with men, sex workers, people who inject drugs and prisoners. Key populations therefore, constitute an important bridging population for HIV transmission to the general population [3]. Progress towards universal coverage of highly active antiretroviral therapy (ART) for PLHIV in SSA has been exponential. Expanded ART initiation criteria for PLHIV over the last decade has been associated with increased longevity and favorable treatment outcomes [4].

Concomitantly, NCDs have risen steadily to become the leading cause of both morbidity and mortality [1]. The Global Burden of Diseases, Injuries and Risk Factors Study (GBD) 2017 indicated a 40% increase between 2007 and 2017 of NCDs disability adjusted life-years (DALYs). NCDs contributed to 62% of total DALYs [5]. That the two epidemics - NCDs and HIV/AIDS are not only a double burden but also on a collision path in sub-Saharan Africa (SSA) is therefore not in question [6]. In fact, some authors have described the current intersection of NCDs and HIV epidemics as a syndemic [7]. Clearly, SSA is undergoing an epidemic transition. For instance, in 2016, Kenya witnessed the entry of two NCDs - ischemic heart disease and cerebrovascular disease, into the top five causes of death [8]. The losses attributed to this dual burden of NCDs and HIV call for an urgent response.

**The global response:** through the Sustainable Development Goal (SDG) 3, the United Nation set ambitious targets to substantially roll back the tide on four main NCDs - cardiovascular diseases, chronic lung diseases, cancer and diabetes mellitus by 2030 [9]. Specifically, SDG 3 target 4 seeks to reduce premature mortality from NCDs by a third through prevention and treatment and promote mental health [9].

**Global action plan and commitment to achieving nine NCD targets:** internationally, the launch of the Global Action Plan (GAP) for the prevention and control of NCDs 2013-2014 served to inject impetus at efforts to control the NCD pandemic. The GAP was a significant endorsement towards concerted efforts aimed at control of NCD pandemic [10]. By implementing the GAP, countries would not only support realization of nine voluntary NCD targets by 2025 but also position themselves to make significant strides towards attainment of SDG 3 on promoting health and well-being for all at all ages. Further, countries signed up to achieving nine voluntary NCD targets by the year 2025 [11]. The main target called for a 25% relative reduction in overall mortality from the four main NCDs (cardiovascular diseases, cancer, diabetes and chronic respiratory diseases), and a similar reduction or containment of the prevalence of raised blood pressure as per national circumstances. The rise of diabetes and obesity were to be halted. Additional targets included achieving at least 10% relative reduction in the use of alcohol, and prevalence of insufficient physical activity. A 30% reduction in mean population intake of salt and a similar reduction in the prevalence of current use in persons 15+ years. The final two targets focused on the health system’s national response. This involved placing at least 50% of eligible people on drug therapy and counseling to prevent heart attacks, and having an 80% availability of affordable basic technology and essential medicines required to treat the major NCDs in both private and public facilities. The nine voluntary targets were underpinned against a global monitoring framework to track implementation of the nine global targets against a 2010 baseline. Countries would set national NCD targets for 2025, develop multi-sectoral national NCD plans with an aim of reducing exposure to risk factors and facilitating a robust health system response and finally measure results against the Global Action Plan [12].

At the 66^th^ United Nations (UN) high level meeting of the general assembly on prevention and control of NCDs, all WHO member states from SSA endorsed the Global Action Plan [13]. A review tracing Africa’s progress towards implementing the NCD GAP 2012-2030 and the WHO’s NCD progress monitor report of 2017 however showed mixed results [14]. Despite many countries making several political commitments towards prevention and control of NCDs, progress has remained largely insufficient and highly uneven [14]. Overall, more than half of African countries had not met their 2015 targets and were making slow progress towards achievement of 2016 targets. Gains were observed in implementation of national campaigns on diet and physical activity. Limited gains were noted for guidelines development for NCD management and drug therapy and counseling. Southern Africa region was least progress while Northern Africa was noted to be the most progressive. Progress in the largest African economies have been remarkable with Nigeria achieving set NCD targets, and instituting demand-reduction measures both harmful alcohol and tobacco use. However, efforts towards guidelines development, drug therapy and public awareness on physical activity have been limited [14]. South Africa on the other hand has been very successful instituting unhealthy diet reduction measures but have had less progress with tobacco and harmful use of alcohol. South Africa has not made progress with public education and awareness campaigns on physical activity [14]. That accelerated action for implementation of the NCD GAP is required is not in question [14]. Beyond political goodwill through declarations, an accountability framework with cost estimates on NCD burden is required. Definition, coordination and characterization of a multi-sectoral approach in developing local policies and programs to address NCD burden would be essential in catalyzing NCD GAP rollout with fidelity [15].

**WHO's 16 best-buys:** the WHO further proposed 16 interventions, referred to as 'best buys', for LMICs’ implementation based on their circumstances. The 'best buys' are the mainstay for WHO’s strategy on NCD control [16]. Using a base year of 2010, these 'best buys' have a potential to avert 9.6 million deaths by 2025 worldwide and 1.13 million deaths from cardiovascular diseases in 20 LMICs [17]. Full intervention of these 'best buys' has the potential to achieve SDG 3.4 on cardiovascular diseases [17]. The 'best-buys' are generally classified by predisposing risk factors of tobacco use, harmful alcohol use, unhealthy diets and physical inactivity. Other interventions address cardiovascular disease, diabetes and cancer. Under tobacco use, four 'best-buy interventions' for LMICs implementation included tax increases, smoke-free indoor workplaces and public spaces, health information and warnings and bans on tobacco advertising, promotion and sponsorship. Interventions under harmful alcohol use included tax increases, restricted access to retailed alcohol and bans on alcohol advertising. To reverse unhealthy diet and physical inactivity, LMICs would implement salt reduction intake in food, replace trans-fat with polyunsaturated fat, and increase public awareness through mass media on diet and physical activity.

Additional interventions to curb cardiovascular disease (CVD) and diabetes would involve counselling and multi-drug therapy for people with a high risk of developing heart attacks and strokes and treatment of heart attacks with aspirin. Lastly, to address cancer, focus would be on Hepatitis B immunization to prevent liver cancer and screening and treatment of pre-cancerous lesions to prevent cervical cancer [18]. Similar to implementation of the NCD GAP, WHO member states’ progress on 'best-buys' implementation is uneven. That the 'best buys' were mainly adopted from high income countries serves to heighten uncertainty over a prioritization index for implementation in the diverse context of LMICs. Between 2000 and 2015, risk of premature death attributed to the four main NCDs declined by 25.4% in high income countries. Such mortality declined only by 7.8% in lower-middle income countries and increased by 6% in low income countries [19]. A 2018 systematic review of 'best-buys' implementation examined the WHO 'best buys' in greater detail [16]. Some 33 LMICs had implemented a national campaign to create public awareness on diet and physical activity [14]. LMICs were found to have no evidence for any of the alcohol 'best-buys' with countries such as Gambia, Ghana, Nigeria and Uganda being aggressively targeted markets. Tobacco use was found to be on the decline in all WHO regions save for Africa.

Further, there was no evidence of five of the six dietary interventions. By 2015, five LMICs had implemented salt reduction policies, but none was a low-income country [14]. There were no African countries found to have evaluated cardiovascular 'best-buys'. Modelling studies however, project that 17.9 million deaths could be averted with cardiovascular polypharmacy at a cost of USD 0.75 - 1.30 per capita. Similarly, for cervical cancer, a single smear at the test age of 40 years with lesion removal and cancer treatment would avert 462 Disability Adjusted Life Years (DALYs) per million people in SSA [20]. Generally, a lack of published evidence of 'best-buy' interventions in LMICs (most studies are in South East Asia) and that some interventions have not even been evaluated serves to dampen the rapid implementation of the WHO 'best-buys' in LMICs [16].

**Package of essential NCD interventions (WHO PEN):** in addition to the best-buys, WHO proposed a package of essential NCD interventions (PEN) for primary health care in low-resource settings [2,21]. The goal of the PEN was to close the gap between what is needed and what is currently available to reduce burden, health-care costs and human suffering due to major NCDs. The PEN outlined strategies to improve equity, efficiency, quality of care and health impact for major NCDs in primary care. Specific interventions were aligned to the WHO health system building blocks of leadership and governance, financing, medical products, health information system, health workforce, and service delivery. Finally, the PEN calls for engagement of communities and empowerment of people for self-care to improve NCD and other health outcomes.

**Tailoring the response to address NCDs among PLHIV:** to achieve gains and favorable NCD care outcomes, LMICs particularly in SSA, need to tailor approaches to their specific country contexts. A looming NCD-HIV syndemic, dwindling donor support for public health programs, and increased clamor for domestic financing are realities that SSA countries will continually need to address. Indeed, SSA is at a watershed point. In this review, we suggest three generic strategies that if contextually adopted would promote and sustain positive health outcomes for NCD care among People living with HIV/AIDS.

**Universal Health Coverage (UHC):** several studies continue to place PLHIV at increased risk for NCD-related morbidity and mortality [22]. Sudden cardiac death is more common in people with HIV [23]. Chronic obstructive pulmonary disease (COPD) doubled the risk of a heart attack among PLHIV. The tension posed by free HIV care services and paid NCD care accentuates poor NCD-HIV care outcomes and disparity. By providing free access to all healthcare, universal health coverage (UHC) promises to turn the tide on NCD-HIV related morbidity and mortality [24]. UHC is encapsulated within SDG 3 target 8 that calls for its achievement by the year 2030. Through UHC are the aspirations of achieving financial risk protection and access to quality healthcare medicines and vaccines [9]. WHO has included UHC as part of its three-prong work program for 2019 - 2023 specifically targeting UHC for an additional one billion people. For LMICs in SSA, achieving UHC is highly desirable. Acceleration by member states to adopting a UHC political declaration is critical. Through UHC, domestic funding for healthcare is likely to increase. Further, UHC promises to catalyze policy environments in many countries to be supportive of health. A health in all policies strategy will advance provision of equitable healthcare and assure access to efficacious and quality care for NCDs. Implementation of the WHO package of essential noncommunicable disease interventions (WHO PEN) within the context of UHC promises substantial reduction of mortality from NCDs. Whilst the attainment of UHC is desirable, maintaining quality is paramount. Healthcare delivery systems in SSA are generally poorly resourced and consequently fragile. Human resources for health are outstretched and below the capita recommendations [25]. Monitoring strategies and support for transition to UHC along a glide path such as through pilot studies and models are necessary to assure high quality service provision.

**Integration: leveraging on the HIV care platform:** healthcare infrastructure and systems for public healthcare delivery in most of SSA remain strained. Contrastingly, owing to sustained global health funding over the last decade, HIV care delivery platforms have grown and are robust. As a response, to deliver NCD care to PLHIV in LMICs, intuitive programmatic thinking calls for leveraging on HIV chronic care platforms [26]. With overall effective delivery of chronic care, HIV platforms present an attractive solution for integrated care delivery [4]. Integration is defined as management and delivery of health services so that clients receive a continuum of preventive and curative services, according to their needs over time and across different levels of the health system. Common models of NCD-HIV integration identified in SSA include integrated community-based screening for HIV and NCDs in the general population; screening for NCDs and NCD risk factors among HIV patients enrolled in care; integration of HIV and NCD care within clinics; differentiated care for patients with HIV and/or NCDs; and population healthcare for all [27].

Benefits from integration of NCDs into existing HIV service delivery platforms would include more affordable health service delivery through leveraging primary vertical platforms to deliver multiple services. Additionally, through a one-stop shop model, we realize both time and cost savings. Other benefits would be reduced duplication and improved cost-efficiency of health workforce, infrastructure, management and financial resources. Finally, country ownership and sustainability would be enhanced through development of country driven and country led health systems for multiple disease burdens [28]. Although integration appears as the instructive strategy for NCD-HIV delivery of services, it may not be a panacea. NCD burden is a much larger and complex challenge requiring multi-stakeholder coordination as well as massive investments into systems. Loading NCD care delivery on existing HIV platforms could result in a weakening of both systems [28]. Despite many attempts at integration, there is a dearth in evidence-based data on integration effectiveness and cost-effectiveness [29]. Surveillance and data collected on NCDs among PLHIV in LMICs lacks the veracity to inform integration of HIV/NCD care models. Evaluating integrated programs is complex with commonly only process and limited outcome measures being available. Few NCD-HIV integrated programs with screening and management approaches that are contextually appropriate for resource-limited settings exist. Gaps remain in literature with even fewer studies on effectiveness, cost, and best practices for integrated chronic care platforms [30].

**Global and regional multi-stakeholder partnerships for institutional response to NCDs:** the NCD-HIV syndemic is a testament of an epidemiologic shift in disease patterns in SSA. There is clear evidence that an effective response will require a coherent response from a global public health partnership from multiple stakeholders including those beyond the health sector [17]. A multi-pronged response that includes societal change towards healthier lifestyle choices, health systems that are re-engineered to address chronic care, fair trade and norms in trade and fiscal decision making are all required. Governments would adopt an all-in health policy environment that would facilitate pre-service training of front-line healthcare workers and managers on holistic NCD-HIV patient care [15]. Higher institutions of learning could add to the response by prioritizing a research agenda that includes tailored approaches to address NCDs in SSA [30]. Similar to the early days of HIV, there is need to involve and engage civil society. Activism from civil society would increase the impetus with which governments develop policies that are responsive to the NCD-HIV syndemic. Additionally, against a backdrop of HIV, engagement with civil society would harmonize a response to the NCD challenge through creation of messaging and strategies around tackling NCDs (Table 1).

**Table 1:** recommendations to tackle NCD burden among people living with HIV in sub-Saharan Africa

**National Implementation of Universal Health Care (UHC)**
National adoption of the UHC political declaration
Catalyse policy environment to support health by increasing domestic healthcare funding
Advance provision of equitable and quality care healthcare for NCDs
**Integration of NCD care into HIV Care and Service Delivery**
Leverage on robust HIV chronic care platforms for integrated care
Foster NCDs screening and surveillance approaches within HIV programs
**Global and Institutional NCD Responses**
Adoption of all-in health policy approach by Government
Pre-service training of frontline healthcare workers on holistic NCD-HIV patient care
Higher Education Institutions prioritizing NCD research agenda
Rights based approach through Civil Society Organizations engagement

## Conclusion

Countries need to make concerted efforts to alter their policy environment and increase domestic resourcing to hasten the response on the NCD-HIV syndemic. Approaches such as integration that seek to leverage on HIV chronic care platforms for delivery of NCD care to PLHIV appear attractive. Universal health care (UHC) promises to inject some impetus in the response but needs an adjunct institutional response. This response would include multiple players beyond the health sector with a governmental coordination mechanism. Priority ought to be placed on a sharpened research focus by higher educational and research institutions on NCD policy and holistic NCD-HIV care. Akin to the HIV epidemic’s narrative, civil society groups in SSA countries need to champion for accelerated action on NCDs and a societal change towards healthier lifestyle choices.
